# Atherosclerosis and Other Related-Arterial Diseases

**DOI:** 10.3390/ijms241310453

**Published:** 2023-06-21

**Authors:** Luis M. Blanco-Colio, Jose L. Martín-Ventura

**Affiliations:** 1 Vascular Reseach Laboratory, IIS-Fundación Jiménez Díaz., Av. Reyes Católicos 2, 28040 Madrid, Spain; 2 CIBERCV, Spain; 3 Medicine Department, School of Medicine, Autonoma University, 28029 Madrid, Spain

Cardiovascular diseases (CVD) are a major cause of morbidity and mortality worldwide, accounting for more than 17 million deaths each year. The term CVD includes different pathologies affecting the heart and circulatory system, such as heart failure, coronary artery disease, stroke, hypertension, atherosclerosis, and aortic aneurysms. These diseases can cause a wide range of symptoms, including chest pain, shortness of breath, fatigue, palpitations, and leg pain. The risk factors for CVD are high blood pressure, high cholesterol levels, smoking, diabetes, obesity, and a family history of CVD. Atherosclerosis is considered the main precursor of CVD and is characterized by pathological remodeling of the vascular wall with the development of atheromatous plaques in the inner lining of medium and large arteries. These plaques can progress and rupture, eventually leading to CV events and death. Early detection and treatment of CVD are critical to improve outcomes and reduce the risk of complications.

This Special Issue entitled “Atherosclerosis and Other Related-Arterial Diseases” of the International Journal of Molecular Sciences includes a number of contributions providing new information about the development and progression of pathological vascular remodeling. 

Accumulation of lipid-laden foam cells is an early step in the pathogenesis of atherosclerosis. The atherogenic oxidized low-density lipoproteins (ox-LDL) are the main sources of accumulating lipids in foam cells. A critical step in foam cell formation is the recognition and internalization of ox-LDL particles by specific macrophage scavenger receptors, including CD36 and scavenger receptor A. The cholesterol accumulation in macrophages increases the expression of ATP-binding cassette transporter A1 (ABCA1), which promotes reverse cholesterol transport, transferring cholesterol from macrophages to the specific lipid-poor cholesterol acceptor, apolipoprotein A1 [1]. In this context, Tachibana et al. [2] describe a new mechanism to modulate ABCA1 expression in macrophages. Fingolimod, an immunosuppressive agent used to treat multiple sclerosis, can reduce atherosclerotic plaque development. Treatment of murine macrophages with fingolimod reduces lipid droplet formation and ABCA1 expression, decreasing lipid accumulation in these cells. These new findings provide evidence for a new mechanism of fingolimod and its possible effects on atherosclerosis.

Leukocyte recruitment to the vessels represents an essential early step in initiating atherosclerosis preceding the local actions of intimal leukocytes. Monocyte-derived macrophages, T lymphocytes, and neutrophils are involved in inflammatory processes inside the vessel wall during atherosclerosis progression [3]. In the vessel, leukocyte recruitment is predominantly achieved by the interaction of endothelial P- and E-selectin with P-selectin glycoprotein ligand-1 and other glycosylated ligands on leukocytes. Focusing on leukocyte adhesiveness to dysfunctional endothelium, López-Riera et al. [4] analyzed the role of the activation of the constitutive androstane receptor (CAR) in the inflammatory response. They demonstrated that the activation of CAR by an agonist induces its nuclear translocation and heterodimerization with the retinoid x receptor α and inhibits the production of tumor necrosis factor (TNF) α-induced cytokines and chemokines in endothelial cells. Furthermore, treatment of mice with the CAR agonist reduced TNF-α-induced leukocyte-endothelial cell interaction, an effect related to decreased VCAM-1 expression within the cremasteric postcapillary venules.

The immune–inflammatory response and vascular remodeling are closely interrelated. Recruitment of immune cells during the inflammatory response can promote vascular remodeling by releasing growth factors and cytokines that stimulate vascular smooth muscle cell proliferation and migration. Conversely, vascular remodeling may contribute to inflammation by altering blood flow and promoting the accumulation of immune cells at the site of injury. The inflammatory response is a complex process that involves multiple cells, signaling pathways, and molecular mediators. Understanding the mechanisms underlying this process is crucial for developing effective therapies against vascular diseases. The review by Martínez-González et al. [5] focused on the role of the nuclear receptor NOR-1 in regulating vascular cells, cardiomyocytes, and inflammatory cells, and the immune response during pathological vascular remodeling. The authors describe the potential role of NOR-1 during atherosclerosis and abdominal aortic aneurysm (AAA) development. In addition, they highlight the contribution of this transcription factor in the activation/proliferation of VSMCs and the vascular neointimal thickening/remodeling demonstrated in different experimental models of vascular injury or pulmonary arterial hypertension.

Vascular calcification is frequently observed in advanced atherosclerotic plaques. Moreover, vascular calcification is highly associated with CVD mortality, particularly in high-risk patients with diabetes and chronic kidney diseases. Intimal calcification is associated with atherosclerosis, whereas medial calcification is a non-occlusive process that results in increased arterial stiffness and reduced vascular compliance. In addition, valve calcification can modify the mechanical properties of the tissue and induce stenosis. Vascular calcification has been related to aging. In recent years, it has been described that vascular calcification is an actively regulated process. Bone-related proteins, including osteocalcin, osteopontin, alkaline phosphate, and Runx2, and matrix vesicles that nucleate hydroxyapatite mineral crystals, have been observed in calcified vascular lesions [6]. Although various mechanisms have been implicated in vascular calcification, our understanding of the pathogenesis of calcification is far from complete. The review performed by Villa-Bellosta [7] focused on the balance between phosphate and pyrophosphate: their control by different genes, and how the imbalance between the concentrations of these two metabolisms plays a key role in vascular calcification. 

Although the inflammatory response and vascular calcification play important roles in the development and progression of atherosclerosis plaques, evidence has accumulated in recent years that indicate the gut microbiota is also implicated in the development of CVD. The composition and functions of the gut microbiome are affected by external factors, including aging, obesity, and dietary patterns. DNA from several bacterial species has been found in atherosclerotic lesions and the intestine of the same individuals, suggesting that the intestinal microbiota could be a potential source of bacteria involved in the development of atherosclerosis [8]. To analyze the role of microbiota in different gut segments, Wen et al. [9] developed an atherosclerotic model in rats fed a high-fat diet for a long period and studied microbiota changes in the intestine. Interestingly, different degrees of alteration of the intestinal microbiota were observed between the ileum and the colon. Higher ileal gut microbiota correlated with lipid levels, intima/media thickness, and atherosclerotic lesion size, whereas colonic gut microbiotas correlated worse with the different parameters analyzed.

3-hydroxy-3-methyl glutaryl-CoA (HMG-CoA) reductase inhibitors (statins) are lipid-lowering drugs used in primary and secondary prevention of coronary heart disease. The main action of statins is to inhibit cholesterol synthesis in the liver by the HMG-CoA reductase. However, muscle pain is one of the most frequent adverse effects in patients taking statins. Therefore, it is important to distinguish patients with statin intolerance before they present muscle symptoms. Mangas et al. [10] identified a new microRNA signature capable of discriminating between statin-intolerant and non-statin-intolerant patients. Moreover, when the authors add clinical information, such as years of dyslipidemia or non-high-density cholesterol levels, this set of circulating miRNAs significantly increases in accuracy.

Finally, in a case report study, Myasnikov et al. [11] demonstrated that double mutation DSG2-p.S363X and TBX20-p.278X is associated with left ventricular non-compaction cardiomyopathy, a rare heart disease characterized by a two-layer structure of the myocardium and increased number of trabeculae. Identifying nonsense variants of these genes could be important for genetic screening of this pathology.

Overall, atherosclerosis is a complex process that involves multiple cellular and molecular mechanisms and is a key contributor to the development and progression of CVD. This collection of articles and reviews provides a current perspective on the role of several mechanisms and potential biomarkers in the development and progression of pathological vascular remodeling.
